# Dark-Chocolate-Coated BRS Clara Raisins: Phenolic Composition and Sensory Attributes

**DOI:** 10.3390/molecules28207006

**Published:** 2023-10-10

**Authors:** Carolina Olivati, Yara Paula Nishiyama-Hortense, Natália Soares Janzantti, Roberto da Silva, Ellen Silva Lago Vanzela, Sergio Gómez-Alonso

**Affiliations:** 1 Institute of Biosciences, Humanities and Exact Sciences (Ibilce), Campus São José do Rio Preto, São Paulo State University (UNESP), Rua Cristóvão Colombo nº 2265, São José do Rio Preto 15054-000, Brazilnatalia.soares-janzantti@unesp.br (N.S.J.); roberto.silva@unesp.br (R.d.S.); ellen.sl.vanzela@unesp.br (E.S.L.V.); 2 Faculty of Chemical Sciences and Technologies, University of Castilla-La Mancha, Avda. Camilo José Cela s/n, 13071 Ciudad Real, Spain; 3 Regional Institute for Applied Scientific Research (IRICA), University of Castilla-La Mancha, Avda. Camilo José Cela s/n, 13071 Ciudad Real, Spain

**Keywords:** raisin, chocolate, dragée, Brazilian cultivar, phenolic composition, HPLC-DAD-ESI-MS/MS

## Abstract

Dark chocolate dragée confectionary was made with BRS Clara raisins pre-treated with extra virgin olive oil (EVOO). The evaluation of the changes in the phenolic composition (flavonols, hydrocinnamic acid derivatives (HCADs), stilbenes and flavan-3-ol monomers, dimers, and proanthocyanidins (PAs)) resulting from the covering process showed that the chocolate coating was responsible for an increase in the concentrations of flavan-3-ols and PAs when compared to just the raisins. For the flavonols and HCADs, a reduction in the total concentration of compounds was observed when comparing the dragées to the raisins. Furthermore, there was a strong influence of chocolate in the qualitative profile with the emergence of new compounds (quercetin-3-pentoside, kampfterol-3-rutinoside, p-coumaric acid, and caffeoyl-aspartate). The combination of these ingredients (raisins and chocolate) resulted in a dark chocolate coated raisin (DC) with good sensory acceptance and a more complex phenolic composition that may positively contribute to its functional quality.

## 1. Introduction

Consumers of different age groups and cultures are increasingly seeking a more mindful approach to their diet. The contribution of healthier snacks to the quality of diet has been highlighted. The development of snacks containing a high concentration of fruit, in particular, is a global market trend due to the growing number of consumers looking for new food products with important nutritional value and high content of compounds with claims to functional properties [1,2,3].

In this context, grapes and raisins are noteworthy for their high concentrations of phenolic compounds (PCs) and the presence of micronutrients, especially minerals and carbohydrates [4]. Raisins offer good satiety compared to other snack options, especially when combined with other ingredients or products, and are extensively utilized across a diverse range of products within the food industry, for example in pannification (cakes, cookies), milk derivatives (yogurt, ice cream), etc. [5,6]. Another product that contains PCs and is widely accepted by consumers is cocoa and its derivatives, such as chocolate [1,7,8,9]. It should be noted that its unique sensory characteristics and functional value are markedly influenced by the composition and concentration of PCs present [10].

Among several products, chocolate-panned confections consisting of a center containing nuts, fruits, parts of fruit, or dried fruits covered by successive layers of a coating, such as chocolate, can be highlighted due to their good acceptance [11]. However, products perceived as too innovative can have a negative impact on liking, even among neophilic consumers [12]. Raisins can be a great center for developing chocolate-panned confections. In Brazil, there are several grape cultivars with potential for processing in the form of raisins, with the emphasis, in this study, on the BRS Clara grape, a seedless white cultivar that presents an interesting composition of PCs and has a pleasant fruity sweet taste [13,14,15].

Some studies have already shown positive changes in the profile of PCs and in the antioxidant activity of products in which raisins [1] and grape pomace [16] were combined with chocolate. Komes et al. (2013) [1], in a study on the influence of the addition of dehydrated fruits (plum, papaya, apricot, raisins, and cranberries) on the concentration of bioactive compounds in dark and milk chocolate, observed that chocolates with raisins had a higher concentration of flavan-3-ols and procyanidins, and also higher antioxidant activity than the control chocolates. Corroborating this study, Acan et al. (2020) [16], in research on the use of dried grape pomace (DGP) as a by-product of wine production in chocolate spread (CS), observed that the final product had a total phenolic content in CS samples ranging from 3415 to 13,754 mg of gallic acid equivalent·kg^−1^, and resveratrol content ranging from 101 to 235 μg·100 g^−1^, which were higher values than the ones for the control group.

There is a need for more detailed scientific study of the individual PCs present in this type of product and how much Brazilian fruits can add functionality and sensory quality to chocolate. Thus, the present study aimed to develop dark chocolate (with high cocoa solids content) panned confections containing raisins produced from the BRS Clara cultivar pretreated with natural surfactant (extra virgin olive oil—EVOO), accelerating the drying process, to obtain a snack with a differentiated qualitative and quantitative PCs composition. To assess the quality of the developed products, the qualitative and quantitative profile of PCs (flavonols, hydrocinnamic acid derivatives (HCADs), stilbenes and flavan-3-ol monomers, dimers, and proanthocyanidins (PAs)) present in the BRS Clara raisins (RCs) and the dark chocolate coated raisins (DCs) was determined using high-performance liquid chromatography (HPLC) coupled to photodiode array and/or mass spectrometry detectors, together with the analysis of acceptance and sensory profile of the DCs using the rate-all-that-apply (RATA) methodology.

## 2. Results and Discussion

The dark-chocolate-coated raisins produced (DC-1 and DC-2) presented adequate visual characteristics for this type of product and a mass gain in relation to the initial center (RCs) as expected, which was approximately 100%. Table 1 shows the basic physical and chemical characteristics of the developed products (DC-1 and DC-2). 

The DCs had moisture content results close to those reported in the literature for other fruits coated with chocolate, such as cherries (8.93% ± 0.01) and figs (7.78% ± 0.01) [11], which allow them good stability over their lifetime. However, to avoid adverse chemical and physical reactions in these products, they need to be packed in dark packages and with limited access to oxygen [11]. The detailed phenolic composition (flavonols, HCADs, stilbenes, and flavan-3-ol monomers, dimers, and PAs) of these RCs and DCs was determined.

### 2.1. Flavonols

In the DC samples, six monoglycosylated flavonols and one free aglycone were detected, totaling seven flavonols (Table 2). 

Among the flavonols found, quercetin pentoside (Q-3-pent) and kaempferol rutinoside (K-3-rut) were detected only in the DCs, not being present in the RCs used as the center of the DCs. It is noteworthy that no trisubstituted B-ring flavonols were detected either. Q-3-pent was identified by means of the ESI-MS/MS signal corresponding to the pseudomolecular ions ([M − H]^−^) of *m*/*z* of 433 and its product ion (*m*/*z* of 301), generated by the loss of a fragment of m/z of 132, corresponding to a lost pentose molecule. 

A significant increase in Q-3-gal and an increase in the absolute mean value for quercetin aglycone (free-Q) were noted when comparing the results for the RC samples to the DC samples (DC-1 and DC-2), which can be attributed to the coating of raisins with chocolate. The literature widely reports the presence of these compounds (Q-3-pent, K-3-rut, and Q-3-gal) in cocoa and its derivatives, even if only in small amounts. Therefore, it can be said that the presence of such compounds in the flavonols profile of the DC samples was a direct contribution of the chocolate used to cover the RCs in the panning process. 

On the other hand, there was a reduction in the total concentration of flavonols in the DCs compared to the values found for the RCs used as the natural center (Table 2), which means that the total concentration of flavonols in the cocoa products used for coating the RCs was lower than that of the RCs. There was also a significant reduction in the molar percentages of Q-3-glcU and Q-3-rut, even though these compounds are normally present in cocoa products such as chocolate. 

Probably, concentrations of Q-3-glcU and Q-rut were higher in the raisin centers (RC) than in the cocoa products used in the panning process, so this process led to a dilution effect of these compounds in the final coated raisins. Another possible explanation for this reduction in the content of Q-3-glcU and Q-rut is the hydrolysis of these flavanols glicosides, due to the conditions of the panning process, to liberate quercetin aglicone (Q). As can be seen in Table 2, the molar ratio of free quecertin in dark-chocolate-coated raisins (DCs) is almost double that of the raisin centers.

To date, detailed phenolic profiles covering the flavonols for cocoa or chocolate products with dehydrated fruits are not known. The existing literature, however, does show that Q-3-pent is the main flavonol in cocoa products, followed by Q-3-glc and smaller amounts of other quercetin and kaempferol derivatives. For example, Oracz et al. (2015) [18], in a study on the effect of the roasting of cocoa beans from five different groups of Theobroma cacao L. on the individual content of flavan-3-ols, anthocyanins, and flavonols, found the following concentration ranges for quercetin-derived compounds: Q-3-pent, 43.32 to 170.82 mg·kg^−1^ on dry defatted weight (ff-DW); Q-3-glc, 34.90 to 125.80 mg·kg^−1^ (ff-DW); Q-3-gal, 9.57 to 33.84 mg·kg^−1^ (ff-DW); and free-Q, 0.60 to 2.79 mg·kg^−1^ (ff-DW).

Andres-Lacueva et al. (2008) [19], in a study with commercial cocoa powder, reported values from 2.10 to 40.33 mg·kg^−1^ for Q-3-pent; from 3.97 to 42.74 mg·kg^−1^ for Q-3-glc; from 0.13 to 9.88 mg·kg^−1^ for Q-3-glcU; and from 0.28 to 3.25 mg·kg^−1^ for free-Q. Martini et al. (2018) [20], in a study of the qualitative and quantitative profile of PCs in dark chocolate (70% cocoa) and dark chocolate enriched with Sakura green tea leaves or turmeric powder, obtained values for Q-3-pent between 28.0 and 41.5 mg·kg^−1^; Q-3-glc between 19.6 and 30.5 mg·kg^−1^; Q-3-gal between 4.9 and 24.4 mg·kg^−1^; Q-3-rut between 0.0 and 42.0 mg·kg^−1^; free-Q between 10.8 and 16.2 mg·kg^−1^; and K-3-glc between 0.0 and 3.7 mg·kg^−1^.

It should be noted that the data referred to in the previous paragraphs are for pure chocolate. In the present study, approximately half of the DC dough is made up of chocolate, and half is made up of raisins. Chocolate is not a rich source of flavonols [21] so it is understandable that the concentration of these compounds determined in the DCs is lower than that determined in just the RCs used as the natural centers.

### 2.2. HCADs 

**Figure 1:**
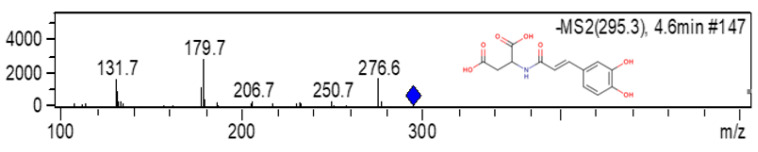
Chemical structure and fragmentation pattern observed for caffeoyl-aspartic acid in dragée samples.

In the DC samples, eight HCADs were detected (Table 2). Of these compounds, six were already present in the RCs (GRP, *trans*-caftaric acid, *cis*- and *trans*-cutaric acids, and *cis*- and *trans*-fertaric acids). The DAHC profile of the DC samples differs from the raisins because of the presence of caffeoyl-aspartic acid, based on the observed fragmentation pattern and using data available in the literature [3,20]. This compound corresponds to the peak at min 4.6 in the 320 nm chromatogram (Figure 1). The presence of this acid has previously been reported in the literature for cocoa powder [3], bitter chocolate (70% cocoa), dark chocolate (70% cocoa and 8% turmeric powder), and dark chocolate (70% cocoa and 2% Sakura green tea) [20]. In addition, the presence of *p*-coumaric acid was also detected in the DC samples, which was identified by detecting its pseudomolecular ion [M-H]- of *m*/*z* 325. These differences between the raisins and the dragée profile reinforce the influence of the dragée process on the phenolic composition of the final product.

The most suitable explanation for the presence of *p*-coumaric acid in DC samples is the hydrolysis of their ester with tartaric acid (coutaric acid) which was originally present in raisins. In fact, the molar ratio of coutaric acid decreased during the panning process while *p*-coumaric acid appeared (Table 2). Coutaric acid is a hydroxycinnamic acid derivative generally present in the skins and pulp of grapes and their hydrolysis during grape processing has been previously described in the literature [22].

*Trans*-caftaric acid was the predominant HCAD in all samples, followed by caffeoyl aspartate in the DC. Even though it was the predominant HCAD in all samples, trans-caftaric acid suffered a significant decrease in its molar proportion. The same behavior was seen with *cis*- and *trans*-coutaric acids and GRP. These results were expected since GRP and tartaric esters of hydroxycinnamic acids are compounds characteristic of grape products, so the reduction in their molar ratios in the DC can be associated with the increase in total weight compared to the raisin weight used as the center. 

As described above for the flavonols, the total concentrations of HCADs determined in the DCs were significantly lower (α = 0.05) when compared to just RCs, since chocolate is not a rich source of this class of compound [21]. In the literature, Martini et al. (2018) [20], when evaluating the qualitative and quantitative profile of PCs in dark chocolate (70% cocoa) and dark chocolate enriched with turmeric powder or Sakura green tea leaves, observed total average contents of HCAD of 1619.5 mg·kg^−1^, 1565.1 mg·kg^−1^ and 1579.8 mg·kg^−1^, respectively.

**Figure 2:**
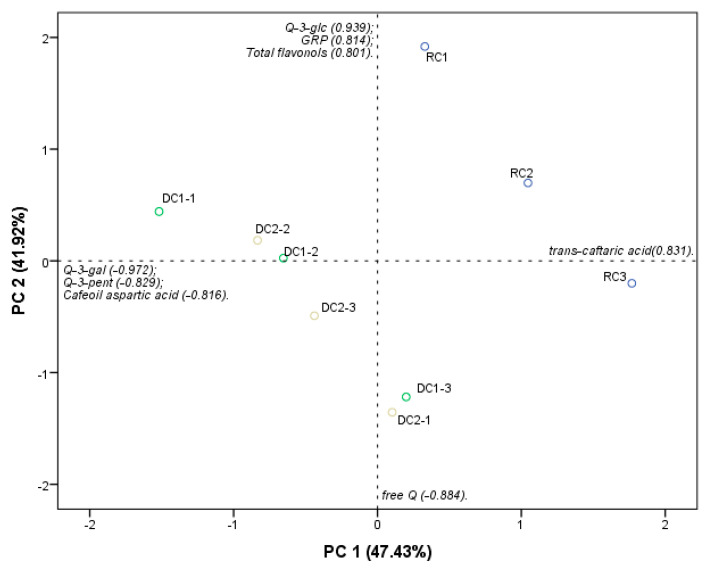
PCA for the flavonols and HCADs of the raisin samples (RCs) and dark-chocolate-coated BRS Clara raisins (DCs).

Principal component analysis (PCA) (Figure 2) reinforces which PCs differentiated RCs (RC-1, RC-2, and RC-3) used as natural centers, from DC (DC1-1, DC1-2, DC1-3, DC2-1, DC2-2, and DC2-3) produced. The DC samples differed from the original RCs in PC 2 (41.9%), characterizing the DCs with higher molar percentages of Q-3-gal, Q-3-pent, and caffeoyl aspartic acid. Caffeoyl aspartic acid and Q-3-pent were found only in the DC samples, not being present in the RCs, therefore being a clear contribution of the chocolate used in the panning to the phenolic profile of the DC. Likewise, the characterization of RCs by their higher molar percentage of *trans*-caftaric acid is consistent with Table 2, since this was the major compound in these samples and was found in higher percentages when compared to those determined in the DCs.

### 2.3. Stilbenes 

In both the DCs and the RCs used as their centers, the *cis*- and *trans*- forms of the compound known as piceid were found, which is a glycoside of resveratrol (Table 3). These compounds, since they are isomers, were differentiated and identified as a function of retention time in the chromatographic column by differentiating their structural forms, the *trans*- compound having a shorter retention time than its respective *cis*- isomer.

The concentration of *trans*-piceid did not show a significant difference between the samples. On the other hand, the content of the *cis*- isomer suffered a significant decrease during the panning process. The maintenance of the concentration of the *trans*-piceid compound in the DC compared to just the raisins can be explained not only by its greater stability compared to its *cis*- isomer but also by being a compound that is present in cocoa and product derivatives such as chocolate [23].

Hurst et al. (2008) [24], when evaluating the levels of trans-piceido in commercial chocolate and cocoa products, found mean concentrations of 1.82 ± 0.36 mg·kg^−^^1^ in dark chocolate and 0.44 ± 0.06 mg·kg^−^^1^ in milk chocolate; higher values than those found for the DCs in this study. As for the other classes of PCs, these differences can, again, be attributed to factors related to the methodologies applied by the studies, genetic and edaphoclimatic influence on the chocolates used, and technological processes involved in the development of the products in focus [25]. Note that the DCs in this study have a proportion of about 50% *w*/*w* of chocolate in relation to the raisins.

### 2.4. Flavan-3-ol Monomers, Dimers, and PA

Five flavan-3-ol monomers ((+)-catechin, (−)-epicatechin), (−)-epigallocatechin, (−)-gallocatechin and (−)-epicatechin 3-gallate) were detected in the samples (Table 4). Among the monomers found, (+)-catechin (C) was the major monomer in the RC, while (−)-epicatechin (EC) was the major monomer in the DC. Panning with chocolate provided a significant increase in all monomers found, except for (−)-epicatechin-3-gallate, since gallolated flavan-3-ols monomers are not usually found in cocoa products so a dilution effect was observed [18,19,20,21].

These results agree with expectations since the literature reports that the class of flavan-3-ols is the main class of PCs for chocolates and cocoa-derived products [19,20,21,25,26,27,28]. It can also be expected, based on the literature, that the main flavan-3-ol monomers for DC will be (−)-epicatechin (EC), followed by (+)-catechin (C), since these are the main flavan-3-ols monomers for chocolates and cocoa products [19,20].

In the DCs evaluated here, EC alone represented about 23% of the total flavan-3-ols found, being the main PC in the products. This is consistent with the literature. EC is reported as the main monomer in cocoa products, representing up to 35% of the total concentration of phenolics, depending on the product and type of processing [20,21,29].

Todorovic et al. (2015) [28], when evaluating the PCs in dark chocolates with raspberries, found that the presence of raspberries did not influence the concentrations of flavan-3-ol monomers, since the mean concentrations of catechin and epicatechin were 137 mg·kg^−1^ and 225 mg·kg^−1^, respectively, for plain dark chocolates, and 176 mg·kg^−1^ and 235 mg·kg^−1^ for dark chocolates with raspberries. The values in that study were higher than those found in the present study, possibly because flavan-3-ols usually concentrate in grape seeds and in grape skins, and the BRS Clara grapes used in this research are seedless.

Other research evaluating the flavan-3-ols profile of chocolates and cocoa products also found higher concentrations. Martini et al. (2018) [20], when evaluating the qualitative and quantitative profile of PCs in chocolate (70% cocoa) and chocolate enriched with turmeric powder or Sakura green tea leaves, observed EC contents of 203.29 ± 10.68 mg·100 g^−1^, 218.43 ± 8.08 mg·100 g^−1^ and 303.69 ± 11.65 mg·100 g^−1^, respectively; C concentrations of 66.20 ± 1.99, 71.13 ± 2.15 and 69.62 ± 5.28 mg·100 g^−1^, respectively; and total flavan-3-ol concentrations of 503.76 ± 8.98, 538.71 ± 8.99 and 726.03 ± 14.53 mg.100 g^−1^, respectively.

Belščak et al. (2009) [27], when evaluating chocolates with different cocoa solid contents in defatted methanol extract, found EC and C values, respectively, of 1.68 ± 0.27 mg·g^−1^ and 0.25 ± 0.17 mg·g^−1^ for chocolate with 88% cocoa solids; 0.75 ± 0.03 mg·g^−1^ and 0.05 ± 0.02 mg·g^−1^ for chocolate with 72% cocoa solids; 1.20 ± 0.20 mg·g^−1^ and 0.59 ± 0.11 mg·g^−1^ for chocolate with 60% cocoa solids; and 0.10 ± 0.01 mg·g^−1^ and 0.02 ± 0.01 mg·g^−1^ for milk chocolate (29% cocoa solids).

Regarding dimers, procyanidins PB1, PB2, and PB4 were detected in all samples analyzed and, for the DCs, PB4 was the major dimer, followed by PB2, while PB1 remained at concentrations statistically the same as that of just the raisins, indicating that cocoa products used in the panning process were a source of PB4 and PB2. PB2 is a procyanidin commonly found in cocoa and cocoa products [30,31,32].

The evaluation of the structural characteristics of non-monomeric flavan-3-ols (PA), that is, from dimers to polymers, was also carried out, through the depolymerization reaction with pyrogallol, releasing a “terminal unit” as flavan-3-ols monomers and the rest of the units, called the “extension unit”, as adducts of flavan-3-ol monomers with pyrogallol (Table 5).

Table 5 shows that, for the DC, the average degree of polymerization (mDP) was approximately 2.70, indicating a predominance of dimer PAs (proanthocyanidins). The percentage of galloylation was significantly lower (α = 0.05) than that found for RC and, in addition, when evaluating the terminal and extension units, there was a prevalence of EC over C in both kinds of unit. Studies in the literature indicate that the structures of cocoa PAs are mainly composed of non-galloylated procyanidins, with a predominance of EC over C [29]. The profile of PAs found for the DCs is closer to that observed for cocoa and cocoa products, thus allowing the conclusion that the addition of chocolate to RC exerted a major influence on the flavan-3-ols and PA profile of the final product.

Regarding the total concentration of PA, there was an increase in the absolute values determined in the DC in relation to just the RC. A similar increase was observed for the analysis of condensed tannins when it was carried out by the methylcellulose method, thus evidencing the greater influence of chocolate against that of RCs in the qualitative and quantitative composition of PA of the final panned confections. Similar results were also found by Todorovic et al. (2015) [28] when evaluating the PCs present in dark chocolates because the presence of raspberries in the dark chocolate did not influence the PA concentration (in mg equivalents of cyanidin chloride.g^−1^—FW), being 2.31 ± 0.80 for plain dark chocolates and 2.27 ± 0.21 for dark chocolates with raspberries.

These results show that the concentration of flavan-3-ols in the DCs produced with RCs was significantly higher due to the contribution of the chocolate. Komes et al. (2013) [1], in a study on the influence of enrichment of chocolates with dehydrated fruits (plums, papaya, apricots, grapes, and cranberries), also observed that the combination of raisins with dark chocolate (42% cocoa solids) resulted in higher concentrations of flavan-3-ols than those found in raisins alone, showing that non-fat cocoa solids were the main source of flavan-3-ols in the products.

**Figure 3:**
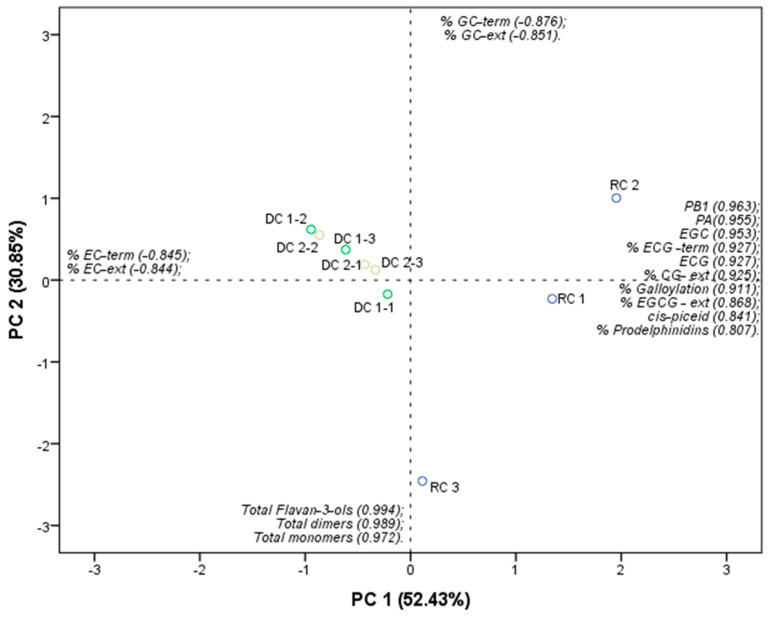
PCA for the stilbenes and flavan-3-ols of the raisins (RC) and dark-chocolate-coated BRS Clara raisins (DC-1 and DC-2).

A PCA for the classes of stilbenes, flavan-3-ols, and PAs (Figure 3), differentiated the DC samples from the RC samples in PC1 (52.43%). 

The DCs were characterized by the % EC-term (−0.845) and % EC-ext (−0.844), illustrating the structural characterization of the PA. Table 5 shows a higher concentration of these compounds in the DC samples. The RC samples were characterized by the percentage concentrations of PB1 (0.963); PA (0.955); EGC (0.953); % ECG-term (0.927); ECG (0.927); % CG-ext (0.925); % Galloylation (0.911); % EGCG-ext (0.868); cis-piceid (0.841); and % Prodelphinidins (0.807), thus illustrating the data presented in Table 4 and Table 5. All these compounds, except ECG, were at a higher concentration in RCs.

It is worth noting that the composition of phenolic compounds in “cocoa products” is strongly affected by factors such as the cocoa variety, the climatic and cultural conditions during harvesting, and the processing methods to which it is exposed. In the case of chocolate, it is also important to take into account the variability introduced by the various stages of chocolate production and the proportions of different ingredients (defatted cocoa powder, cocoa butter, milk solids, sugar, etc.) [33,34]. 

### 2.5. Sensory Analysis 

The RATA technique together with acceptance allows the characterization of a new product using untrained consumers and an assessment of its acceptance [35].

Table 6 shows the average of the sensory descriptors and the acceptance for the DCs. The DCs were described (on a scale between 4 and 5) mainly by the terms brown color and soft, in addition to the hedonic terms tasty, pleasant, interesting, and delightful. It is worth mentioning that the descriptors brown color, sweet taste, and soft texture, in addition to the hedonic terms delicious and delightful, were considered applicable by all consumers who evaluated the DCs.

The majority of consumers (82%) declared that they liked very much or liked extremely (hedonic values between 8 and 9) the DCs, which is clearly reflected in the mean global acceptance of the product (8.20 ± 0.89) (Table 6). No consumer declared disliking the DCs; that is, all consumers positively evaluated the product (hedonic values above 6, slightly liked term), thus showing the excellent acceptance of the DCs by consumers.

Komes et al. (2013) [1], in the sensory analysis of dark chocolate and milk chocolate, added dehydrated fruits (plum, papaya, apricot, raisins, and cranberries) to improve bioactive compounds, and observed that the highest overall acceptability was recorded for milk chocolate with dried cranberries, followed by plain milk chocolate and milk chocolate with raisins. Among bitter chocolates, plain bitter chocolate scored highest in terms of acceptability, followed closely by chocolate with dried apricots. These results show that the overall acceptability is influenced by both the sort of fruit and the type of chocolate.

With the aim of verifying which sensory descriptor evaluated had a correlation with the global acceptance of the dragées, and whether there was a correlation between the terms used, a Pearson Correlation Analysis was carried out with a 95% confidence level (Table S1).

The global acceptance of the dragée had a moderate correlation with the descriptor “soft” of the texture attribute (0.440) and with the hedonic terms tasty (0.380), pleasant (0.289), natural (0.301), interesting (0.382), and delightful (0.366). Among the descriptor terms, it was possible to verify a strong correlation (*p* ≤ 0.01) between the odor and flavor of milk chocolate (0.699) and between the hedonic terms interesting and delightful (0.764), tasty and pleasant (0.751), and pleasant and delightful (0.606).

Negative correlations were found between milk chocolate flavor and dark chocolate flavor (−0.341), fat/cocoa butter odor and dark chocolate odor (−0.343), and the hedonic term delightful and the taste of fat/cocoa butter/butter (−0.303). These correlations allow us to note that consumers were able to discriminate between milk chocolate flavor and dark chocolate flavor, and between dark chocolate odor and fat/cocoa butter/butter odor.

Furthermore, it was also possible to note that the consumers questioned did not appreciate/expect the fat/cocoa butter/butter flavor in this type of product, and that the presence of this flavor would make the product less pleasurable in the consumer’s perception. Together, the results of this study and the literature state that the combination of chocolate with dried fruits is not just interesting but also likable for the consumer in general.

Other studies have confirmed that a chocolate coating has a significant impact on sensorial characteristics, suggesting the potential for creating novel products that could achieve widespread consumer acceptance in the market. For example, Yeganehzad et al. (2020) [9] reported that samples coated with chocolate scored higher (for appearance, flavor, texture, and total acceptance) than the core sample only. Likewise, Tassi et al. (2019) [35] showed that roasted black soybeans with a chocolate coating were well accepted by young tasters, even if they did not have a habit of consuming soybeans.

## 3. Conclusions

After pre-treatment of the RCs with EVOO and then a chocolate coating, the mixture of chocolate and RCs resulted in a product with a more complex profile and a high concentration of PCs, observed through the emergence of compounds that were not present in the RCs (quercetin-3-pentoside, kampferol-3-rutinoside, *p*-coumaric acid, and caffeoyl-aspartate). For the flavan-3-ols, it was possible to notice the great contribution of the chocolate applied as a coating in the total content of this class of PC once the concentration of flavan-3-ols in the DCs produced with RCs was significantly higher, from approximately 48 mg eq. of C kg^−^^1^ DW in the raisins to approximately 515–517 mg eq. of C kg^−^^1^ DW in the dragées. This was also observed for the PA, with an increase from approximately 422 mg·kg^−^^1^ of product—DW in the raisins to approximately 768–783 mg·kg^−^^1^ of product—DW in the dragées. Furthermore, all consumers positively evaluated the product during sensory analysis (82%). 

These results, together, indicate that DCs are an attractive product for consumers and a possible source of PCs. Moreover, this study provided a wide PC profile for a chocolate-coated fruit product, increasing knowledge and providing background information for future studies. Based on the findings of the study, several suggestions for future research or areas of investigation can be proposed, such as bioavailability studies, antioxidant activity, storage stability, and health benefits that could contribute to a deeper understanding of the phenolic composition, sensory attributes, and potential health effects of chocolate-coated products, as well as guide the development of innovative and nutritious food products.

## 4. Materials and Methods

### 4.1. Chemicals

The chemical standards caffeic acid, *p*-coumaric acid, *trans*-caftaric acid, *trans*-piceid, (−)-epigallocatechin (EGC), (−)-gallocatechin (GC), and procyanidin B1 (PB1) (Phytolab, Vestenbergsgreuth, Germany); cyanidin-3-glucoside (cy-3-glc), procyanidin B2 (PB2), quercetin (Q), kaempferol (K), isorhamnetin (I), the 3-glucosides of Q, K, I and 3-galactosides of Q, K, and I (−)-catechin 3-gallate (CG), (−)-epicatechin 3-gallate (ECG), and (−)-epigallocatechin 3-gallate (EGCG) (Extrasynthese, Genay, France); gallic acid, *trans*-resveratrol, (+)-catechin (C), (−)-epicatechin (EC), and (−)-gallocatechin 3-gallate (GCG) (Sigma-Aldrich, Steinheim, Germany) were used. The non-commercial flavonol standard of quercetin 3-glucuronide (Q-3-glcU) was previously isolated from the skin of Petit Verdot grapes, and the Procyanidin B4 (PB4) was kindly supplied by the Department of Biochemistry and Biotechnology, Universitat Rovira i Virgili, Spain. The *trans-* isomers of resveratrol and its 3-glucosides (piceid) were obtained according to the methodology previously described [36]. All solvents used were chromatographic grade (>99%); the chemical standard was analytical grade (>95%) and ultrapure water (Milli-Q system) was used.

### 4.2. Ingredients for the Dark-Chocolate-Panned Confections 

Ripe BRS Clara grapes were sourced from the city of Marialva (Paraná State, Brazil) while ensuring good sanitary conditions. EVOO was purchased from the local market and used as a natural surface-active agent for the grape pre-treatment in raisin production. The EVOO had a maximum acidity ≤ 0.50% and peroxide index ≤ 20.00 mEq O2·kg^−1^. The necessary ingredients for the preparation of the dark-chocolate-panned confections were acquired from specialized suppliers: gum arabic type 4880 (Willy Benecke, Vogler Ingredients); pure cocoa powder (Nestlé^®^); and dark chocolate (with at least 35% cocoa mass) (Melken—Gotas—Harald^®^).

### 4.3. Production of Raisin Centers

Raisins were prepared from the BRS Clara grapes. First, the grapes were submitted to the pre-treatment with EVOO and dried according to the methodology previously described in the literature [15,36]. In order to minimize the migration of moisture from the center to the surface of the dark chocolate, the raisins were produced with an average moisture value of 13%. The two portions of raisins were sealed using a solution composed of gum arabic and water (4:6, *m*/*m*), followed by coating with cocoa (100%) to make the surface of the raisins smoother and more uniform, thus improving the adherence of the chocolate. The sealed raisins were then placed in shallow trays and kept at rest for 24 h in a dry environment until used as centers in the panning process.

### 4.4. Chocolate Dragée Production

For the preparation of the dragées, the RC centers were covered with dark chocolate previously melted at 40–45 °C in a bain marie. This procedure was carried out in an IBD-10 benchtop coating machine (InoxTech, São Paulo, Brazil), where approximately 25 g portions of melted chocolate were manually poured over the RCs with a ladle and mixed in the coating machine until all were covered and the chocolate was dry. The number of portions of chocolate added was standardized as that which formed a smooth and uniform chocolate layer that provided a mass gain between 90% and 100%, based on the mass of the RCs. This process was conducted twice, in order to obtain two batches of DCs (DC-1 and DC-2), and thus verify the reproducibility of the process. The final products (DC-1 and DC-2) were stored in polyethylene containers under cooling (±10 °C) until used. The physical and chemical characteristics of the panned confections were determined according to official methods of analysis [37]: moisture (%), using the thermogravimetric method; hydrogen potential (pH), measured directly in a pH meter; and total acidity (TA) (as tartaric acid g·100 g^−1^). In addition, the mass (g) was obtained by weighing the dragées on an analytical scale, and the length (L, cm) and width (W, cm) were measured using a caliper.

### 4.5. Identification and Quantification of PCs of RC and DC Using HPLC-DAD-ESI-MS/MS

To analyze the comprehensive phenolic composition of the RCs and the DC-1 and DC-2, the targeted compounds were initially extracted utilizing established methods [14,36] with slight modifications. All procedures were performed in triplicate. First, the samples were crushed under freezing conditions in an A10 basic mill (IKA, Staufen, Germany) along with solid CO_2_ until a powdered state was achieved. Subsequently, phenolic extracts were generated from 10 g portions of powdered samples following the methods outlined by Olivati et al. (2019) [36]. 

For the dragées, after obtaining the phenolic extracts of the DC, they were defatted by means of a liquid–liquid extraction with hexane at the rate of 10 mL of hexane for every 25 mL of fat extract. The extract–hexane mixture was submitted to an ultrasonic bath (25 °C for 5 min), and the phases were separated by centrifugation (4 °C, 5640 g for 3 min). This procedure was performed three more times with the lower part of the biphasic solution, in order to guarantee the total degreasing of the extract. 

Each extract obtained from the samples (grapes in natura, raisins, and dragées) produced was rotoevaporated (Model Hei-Vap Advantage, Heidolph, Schwabach, Germany) at 37 °C for the elimination of methanol and standardization of the volume up to 50 mL. The extracts were packed in amber glass flasks.

The separation, identification, and quantification of flavonols and HCADs of the RC and DC extracts were carried out using an Agilent 1100 Series HPLC system (Agilent Technologies, Santa Clara, CA, USA) equipped with a Diode Array Detector (DAD; G1315B) and a liquid chromatograph/mass selective detector (LC/MSD; Trap VL G2445C VL) electrospray ionization mass spectrometry (ESI-MS^n^) system, coupled to an Agilent ChemStation (version B.01.03) data-processing unit. The mass spectra data were processed using the Agilent LC/MS Trap software (version 5.3). 

Aliquots of RC and DC extracts underwent solid-phase extraction using Bond Elut Plexa PCX cartridges (6 cm^3^, 500 mg of adsorbent) (Agilent Technologies, Santa Clara, CA, USA). The extracts were then filtered through a Chromafil PET 20/25 polyester membrane (0.20 mm) (Macherey-Nagel, Düren, Germany), and subsequently injected (20 µL) directly into a Zorbax Eclipse XDB-C18 reversed-phase column (2.1 mm × 150 mm; 3.5 µm particle) (Agilent Technologies, Santa Clara, CA, USA). The column was maintained at 40 °C, following the method outlined by Rebello et al. (2013) [38].

For PCs identification, a turbo spray ionization mass spectroscopy system ESI/MS-MS ion trap analyzer was used in negative ionization mode (for flavonols and HCADs), following the method previously described by Rebello et al. (2013) [38]. 

The identification primarily relied on spectroscopic data (UV–Vis and MS/MS) obtained from authentic standards or information sourced from previous studies [14,37,38]. In quantifying PCs, DAD-chromatograms were extracted at 360 nm (for flavonols) and 320 nm (for HCADs). Standards were employed for both identification and quantification, utilizing calibration curves encompassing the anticipated concentration ranges. In cases where standards were not available, quantification was performed by employing the calibration curve of the most similar compound: for the flavonol 3-glycosides and their free aglycones, the standard used was Q-3-glc; and for HACDs, the standard used was caftaric acid.

The stilbene, flavan-3-ol monomer, and proanthocyanidin (PA) analyses were carried out using an HPLC Agilent 1200 series system coupled to an AB Sciex 3200 QTRAP (Applied Biosystems, Foster City, CA, USA) with an ESI-MS/MS system operating in Multiple Reaction Monitoring (MRM) mode. The chromatographic system was managed by an Agilent ChemStation (version B.01.03) data-processing unit, and the mass spectra data were processed using the Analyst MSD software, version 1.5 (Applied Biosystems, Foster City, CA, USA), as previously described by Rebello et al. (2013) [38]. Compounds were separated in an Ascentis C18 reversed-phase column (150 mm × 4.6 mm with 2.7 μm of particle size) (Sulpeco, Germany), with the temperature kept at 16 °C. Initially, aliquots of the phenolic extracts from the samples (RCs and DCs) were subjected to solid-phase extraction using Sep-pak Plus C18 cartridge files (with 820 mg of adsorbent) (Waters Corp., Milford, CT, USA). The approach was in accordance with the protocol delineated by Rebello et al. (2013) [38]. Subsequently, these samples underwent a depolymerization reaction, facilitated by pyrogallol acting as a nucleophilic trapping agent, followed by injection (30 μL).

The structural details of the PA were ascertained using the acid catalysis method involving depolymerization induced by pyrogallol [39]. All established standards were employed for both identification and quantitation, utilizing calibration curves covering the expected concentration ranges. In cases where standards were unavailable, the quantitation was conducted using the calibration curve of the most similar compound: for total polymeric flavan-3-ols (total PA), the quantitation was performed using (+)-catechin (C) as standard; and the total sum of flavan-3-ol monomers and dimers was calculated considering mg of C equivalents. The results were expressed as mean value ± standard deviation in dry weight (DW) and fresh weight (FW).

### 4.6. Sensory Analysis

Forty-nine consumers, aged between 18 and 34 years old, who liked and consumed raisins and dark chocolate, evaluated the DCs through an acceptance test and descriptive analysis using the RATA method [40]. In the acceptance test [41], the three-digit number-encoded DCs were evaluated by consumers using a structured nine-point hedonic scale (1—I really disliked it; 5—I neither liked it nor disliked it; 9—I liked it very much).

In RATA, the consumer was instructed to indicate the intensity (0—not applicable; 1—slightly applicable; 5—very applicable) of each of the descriptors for the dragée, coded by a three-digit number. The sensory descriptors (3 for appearance, 3 for odor, 3 for taste, 4 for flavor, 2 for texture, and 5 for hedonic terms) were developed by a descriptive team. The descriptors presented were randomized [42].

Consumers performed sensory analyses in individual booths lit by incandescent light. The study was approved by the local research ethics committee under the Certificate of Presentation for Ethical Consideration (CAAE) nº 72414717.1.0000.5466.

### 4.7. Data Analysis 

The results of the physical and chemical analyses of the DCs were submitted to the t-test for comparison of means of independent samples with a significance level of 5% (α = 0.05). The results of the chromatographic analyses (flavonols, DAHC, stilbenes, flavan-3-ols, and PAs) for all samples (RCs and DCs) were submitted to analysis of variance (ANOVA) and mean comparison using the Student–Newman–Keuls test, with a significance level of 5% (α = 0.05). A principal component analysis (PCA) was used with the chromatographic results of the DCs in order to identify compounds, based on the qualitative and quantitative profile of the PCs, which differentiate the samples studied from each other. For the construction of the graphs, the principal components that allowed a better separation between the samples were chosen. The test considered the variables (the principal component of each chemical class, as well as the total concentrations for each class) most correlated with each principal component (“loadings” in the “Rotated Component Matrix” > 0.80). Therefore, after the name of the PC, its acronym was described in parentheses followed by the value of “loadings”. Pearson’s linear correlation analysis was performed between the results of sensory analysis, considering a significance level of *p* ≤ 0.05 and correlation r ≥ 0.289. All analyses were performed using the IBM SPSS Statistics V 20.0 software (SPSS Inc., IBM, Armonk, NY, USA).

## Figures and Tables

**Table 1:** Physical and chemical characteristics of the dark-chocolate-coated BRS Clara raisins (DCs) (^3^ mean ± standard deviation, *n* = 3).

Parameter	DC-1	DC-2
Length (cm)	2.25 ± 0.13	2.13 ± 0.04
Width (cm)	1.38 ± 0.17	1.58 ± 0.17
Mass (g) ^1^	2.66 ± 0.17	2.91 ± 0.55
pH	4.45 ± 0.11	4.53 ± 0.07
Acidity ^2^	1.23 ± 0.15	1.19 ± 0.04
Moisture % (*m*/*m*)	9.16 ± 0.00	9.14 ± 0.00

^1^ Abbreviations: cm, centimeters; g, grams. ^2^ Acidity in g tartaric acid·100 g^−1^ of product. ^3^ Results between themselves showed no significant difference (Student–Newman–Keuls test, α = 0.05). Adapted from Olivati, 2020 [17].

**Table 2:** Spectral characteristics (MS/MS) of flavonols and DAHC, identified in the raisin samples (RC) and dark-chocolate-coated BRS Clara raisins (DC-1 and DC-2) by HPLC-DAD-ESI-MS/MS (negative ionization mode), molar ratios (mean value ^2^ ± standard deviation, *n* = 3) and total concentration (expressed in mg eq. of Q-3-glc·kg^−1^ of product and mg eq. of caftaric acid·kg^−1^ of product, respectively).

**Flavonols**	**Deprotonated Molecule and Product Ions (*m*/*z*)**	**Molar Ratios (%) ^1^**		
		**RC**	**DC-1**	**DC-2**
Q-3-gal	463; 301	3.39 b ± 0.90	5.70 a ± 0.75	5.23 a ± 0.44
Q-3-glcU	477; 301	48.97 a ± 3.13	24.05 b ± 0.97	24.08 b ± 1.53
Q-3-glc	463; 301	36.15 a ± 4.30	30.52 a ± 1.49	29.44 a ± 2.13
Q-3-rut	609; 301	4.17 a ± 0.04	2.07 b ± 0.19	1.81 b ± 0.31
Q-3-pent	433; 301	ND	23.00 a ± 1.35	21.64 a ± 1.53
K-3-rut	593; 285	ND	2.19 a ± 0.35	1.95 a ± 0.22
free Q	301	7.32 a ± 2.03	12.47 a ± 4.50	15.85 a ± 5.67
Total (mg·kg^−1^, DW)		60.96 a ± 28.35	32.92 a ± 10.73	34.65 a ± 7.16
Total (mg·kg^−1^, FW)		54.84 ± 8.89	28.33 ± 9.18	29.32 ± 5.76
**HCAD**	**Deprotonated molecule and product ions (*m*/*z*)**	**RC**	**DC-1**	**DC-2**
GRP	618; 543; 489; 264	8.82 a ± 1.57	5.13 b ± 1.18	5.10 b ± 1.16
*trans*-caftaric acid	311; 179; 149; 135	69.40 a ± 4.14	28.29 b ± 4.72	29.59 b ± 2.58
cafeoil aspartic acid	294; 179; 131	ND	26.32 a ± 1.00	24.58 a ± 2.94
*trans*-coutaric acid	295; 163; 149; 119	7.62 a ± 0.86	3.41 b ± 0.36	3.61 b ± 0.25
*cis*-coutaric acid	295; 163; 149; 120	5.41 a ± 0.76	2.51 b ± 0.19	2.61 b ± 0.10
*trans*-fertaric acid	325; 193; 149	5.51 b ± 0.93	14.17 a ± 2.53	13.06 a ± 1.40
*cis*-fertaric acid	325; 193; 149	3.23 a ± 0.33	2.60 a ± 1.30	2.27 a ± 0.30
*p*-coumaric acid	325; 163, 145, 119, 117	ND	17.56 a ± 3.98	19.17 a ± 5.27
Total DAHC (mg·kg^−1^, DW)		285.67 a ± 28.02	128.04 b ± 73.57	107.84 b ± 51.15
Total DAHC (mg·kg^−1^, FW)		129.36 ± 71.31	73.12 ± 44.40	62.16 ± 30.10

Abbreviations: DW, dry weight; FW, fresh weight; glcU, glucuronide; gal, galactoside; glc, glycoside; K, kaempferol; GRP, 2-S-glutathiol-caftaric acid; ND, undetectable; Q, quercetin; rut, rutinoside (6″-rhamnosylglucoside); ^1^ Calculated molar ratios totaling 100%. ^2^ “a” and “b” different letters on the same line indicate a significant difference (Student–Newman–Keuls test, α = 0.05). Adapted from Olivati, 2020 [17].

**Table 3:** Spectral characteristics (MS/MS) of stilbenes identified in the samples of raisins (RCs) and dark-chocolate-coated BRS Clara raisins (DC-1 and DC-2) (mean value ± standard deviation, *n* = 3).

Stilbenes	*m*/*z* Pairs	mg·kg^−1^ of Product—DW *(mg·kg^−1^ of Product*—*FW)*		
		RC	DC-1	DC-2
*trans*-piceid	389/227; 389/185	0.59 a ± 0.03 *(0.51 ± 0.02)*	0.40 a ± 0.13 *(0.36 ± 0.12)*	0.39 a ± 0.02 *(0.35 ± 0.02)*
*cis*-piceid	389/227; 389/185	0.67 a ± 0.01 *(0.59 ± 0.01)*	0.17 b ± 0.02 *(0.16 ± 0.01)*	0.18 b ± 0.01 *(0.16 ± 0.00)*
Sum of *cis*- and *trans*-piceid (mg eq. of *trans*-piceid·kg^−1^ of product)		1.26 a ± 0.04 *(1.10 ± 0.04)*	0.57 b ± 0.15 *(0.52 ± 0.14)*	0.57 b ± 0.01 *(0.52 ± 0.01)*

Abbreviations: DW, dry weight; FW, fresh weight; “a” and “b” different letters on the same line indicate a significant difference (Student- Newman-Keuls, S-N-K test, α = 0.05). Adapted from Olivati, 2020 [17].

**Table 4:** Spectral characteristics (MS/MS) of flavan-3-ols monomers and dimers (type B procyanidins) identified in the samples of raisin (RC) and dark-chocolate-coated BRS Clara raisins (DC-1 and DC-2) (mean value ± standard deviation, *n* = 3).

**Flavan-3-ols Monomers**	***m*/*z* Pairs**	**mg·kg^−1^ of Product—DW** ***(mg·kg^−1^ of Product—FW)***		
		**RC**	**DC-1**	**DC-2**
(+)-catechin	289/137; 289/164	10.12 b ± 5.02 *(8.77 ± 4.34)*	60.23 a ± 8.60 *(54.71 ± 7.82)*	57.42 a ± 1,90 *(52.17 ± 1.73)*
(−)-epicatechin	289/137; 289/164	3.46 b ± 1.89 *(3.00 ± 1.64)*	117.86 a ± 13.80 *(107.06 ± 12.53)*	117.66 a ± 4.55 *(106.90 ± 4.13)*
(−)-galocatechin	305/109; 305/137	0.40 a ± 0.10 *(0.35 ± 0.09)*	0.38 a ± 0.08 *(0.35 ± 0.07)*	0.30 a ± 0.09 *(0.27 ± 0.08)*
(−)-epigalocatechin	305/109; 305/137	0.16 b ± 0.10 *(0.14 ± 0.09)*	0.30 b ± 0.13 *(0.27 ± 0.12)*	0.52 a ± 0.09 *(0.47 ± 0.08)*
(−)-epicatechin-3-galate	441/245; 441/289	0.87 a ± 0.18 *(0.76 ± 0.15)*	0.14 b ± 0.05 *(0.13 ±0.05)*	0.17 b ± 0.03 *(0.16 ± 0.03)*
Total monomers		21.19 b ± 11.70 *(18.37 ± 10.12)*	253.24 a ± 29.70 *(230.05 ± 26.98)*	251.13 a ± 5.10 *(228.17 ± 4.64)*
**Flavan-3-ols dimers**	***m*/*z* pairs**	**mg·kg^−1^ of product—DW** ***(mg·kg^−1^ of product—FW)***		
		**RC**	**DC-1**	**DC-2**
Procianidin B1	577/425; 577/407	19.06 a ± 7.04 *(16.53 ± 6.08)*	10.16 a ± 2.61 *(9.23 ± 2.38)*	10.37 a ± 1.16 *(9.43 ± 1.06)*
Procianidin B2	577/425; 577/407	10.06 b ± 6.19 *(8.72 ± 5.36)*	208.76 a ± 30.31 *(189.65 ± 27.53)*	209.74 a ± 12.31 *(190.57 ± 11.18)*
Procianidin B4	577/425; 577/407	11.94 b ± 7.30 *(10.35 ± 6.36)*	247.76 a ± 35.97 *(225.07 ± 32.68)*	248.92 a ± 14.61 *(226.16 ± 13.27)*
Total dimers		26.80 b ± 12.72 *(23.23 ± 10.99)*	263.50 a ± 39.90 *(239.37 ± 36.25)*	263.83 a ± 15.37 *(239.71 ± 13.97)*
Sum of monomers and dimers (mg eq. of catechin·kg^−1^ of product)		47.99 b ± 24.36 *(41.60 ± 21.07)*	516.73 a ± 68.70 *(469.41 ± 62.41)*	514.97 a ± 20.34 *(467.88 ± 18.49)*

Abbreviations: DW, dry weight; FW, fresh weight; “a” and “b” different letters on the same line indicate a significant difference (S-N-K test, α = 0.05). Adapted from Olivati, 2020 [17].

**Table 5:** Structural characterization of proanthocyanidins (PAs) (mean value ^2^ ± standard deviation, *n* = 3) of raisins (RCs) and dark-chocolate-coated BRS Clara raisins (DC-1 and DC-2).

PA ^1^	RC	DC-1	DC-2
mDP	4.15 a ± 0.31	2.70 b ± 0.12	2.71 b ± 0.02
% galoylation	5.90 a ± 0.94	0.51 b ± 0.16	0.42 b ± 0.01
% prodelphinidin	6.76 a ± 0.47	2.24 b ± 0.73	2.08 b ± 0.21
% C-terminal	49.03 a ± 3.54	23.75 b ± 0.99	22.86 b ± 0.29
% EC-terminal	16.21 b ± 1.64	46.55 a ± 0.67	46.84 a ± 1.01
% GC-terminal	2.60 a ± 2.35	0.14 a ± 0.03	0.11 a ± 0.03
% EGC-terminal	0.69 a ± 0.15	0.12 b ± 0.06	0.20 b ± 0.03
% ECG-terminal	3.14 a ± 1.46	0.03 b ± 0.01	0.04 b ± 0.01
% C-extension	3.15 b ± 0.51	4.86 a ± 0.76	4.72 a ± 0.25
% EC-extension	84.63 b ± 1.53	91.58 a ± 1.95	92.22 a ± 0.47
% EGC-extension	7.18 a ± 0.39	2.99 b ± 1.03	2.60 b ± 0.31
% CG-extension	4.54 a ± 0.94	0.51 b ± 0.15	0.41 b ± 0.01
% GC-extension	0.01 a ± 0.01	0.01 a ± 0.00	0.01 a ± 0.00
% ECG-extension	trace	trace	trace
% GCG-extension	ND	0.01 a ± 0.00	0.01 a ± 0.00
% EGCG-extension	0.49 a ± 0.04	0.04 b ± 0.02	0.03 b ± 0.01
Total PA ^2^ mg·kg^−1^ of product —DW (*mg·kg^−1^ of product —FW*)	421.65 a ± 137.60 *(365.62 ± 118.61)*	768.32 a ± 208.22 *(697.96 ± 189.15)*	783.14 a ± 57.57 *(711.54 ± 52.30)*

^1^ Abbreviations: MDP, medium degree of polymerization; % Galloylation, % 3-gallate units; % Prodelphinidins, % epigallocatechin; ND, undetectable. ^2^ a, b: different lowercase letters on the same line indicate significant differences in concentration for samples (S-N-K test, α = 0.05). Adapted from Olivati, 2020 [17].

**Table 6:** Means (±standard deviation, *n* = 49) attributed to sensory descriptors, hedonic terms, and acceptance for the dark-chocolate-coated BRS Clara raisins.

Sensory Analysis		Mean ± Standard Deviation
Sensory descriptors ^1^		
Appearance	Brown color	4.57 ± 0.87
	Opacity	3.71 ± 1.35
	Icing roughness	1.65 ± 1.51
Odor	Dark chocolate	3.33 ± 1.08
	Milk chocolate	2.47 ± 1.71
	Fat/Cocoa butter	2.65 ± 1.73
Taste	Sweet	3.86 ± 1.08
	Sour	2,75 ± 1.81
	Bitter	1.88 ± 1.54
Flavor	Dark chocolate	2.71 ± 1.65
	Milk chocolate	2.90 ± 1.61
	Raisins	3.88 ± 1.45
	Fat/Cocoa butter/Butter	2.12 ± 1.70
Texture	Soft	4.20 ± 0.96
	Adhesive	2.02 ± 1.70
Hedonic Terms	Tasty	4.37 ± 0.88
	Pleasant	4.18 ± 1.15
	Natural	2.98 ± 1.73
	Interesting	4.14 ± 1.32
	Delightful	4.31 ± 1.08
Acceptance ^2^		8.20 ± 0.89

^1^ 0—not applicable; 1—hardly applicable; 5—very applicable; ^2^ 1—I disliked very much; 5—neither liked nor disliked; 9—I really liked it. Adapted from Olivati, 2020 [17].

## Data Availability

The data presented in this study are available on request from the corresponding author.

## Supplementary Materials

The following supporting information can be downloaded at: https://www.mdpi.com/article/10.3390/molecules28207006/s1, Table S1. Pearson Correlation between the sensory descriptors and the acceptance of DG.

## Abbreviations

C, (+)-catechin; CG, (−)-catechin 3-gallate; cmglc, 6″-(p-coumaroyl)glucoside; DAD, diode arrange detector; DC, dark chocolate coated raisin; DW, dry weight; EC, (−)-epicatechin; ECG, (−)-epicatechin 3-gallate; EGC, (−)-epigallocatechin; EGCG, (−)-epigallocatechin 3-gallate; EMBRAPA, Brazilian Agricultural Research Corporation; eq., equivalents; ESI-MSn, electrospray ionization mass spectrometry system; EVOO, extra virgin olive oil; ext, extension unit; ff-DW, dry defatted weight; FW, fresh weight; g, grams; gal, galactoside; GC, (−)-gallocatechin; GCG, gallocatechin 3-gallate; glc, glucoside; glcU, glucuronide; GRP, 2-S-glutationil-trans-caftaric; HCADs, hydroxycinnamic acid derivatives; HPLC, high performance liquid chromatography; I, isorhamnetin; K, kaempferol; kg, kilograms; L, length; m, meters; mDP, mean degree of polymerization; MRM, Multiple Reaction Monitoring; ND, undetectable; PA, proanthocyanidin; PC, principal component; PCA, principal component analysis; PCs, phenolic compounds; pH, hydrogen potential; Q, quercetin; RATA, rate-all-that-apply; RC, BRS Clara raisin; rut, rutinoside (6″-rhamnosylglucoside); s, seconds; term, terminal unit; TA, total acidity; W, width.
