# Supplementary material for: Dark-Chocolate-Coated BRS Clara Raisins: Phenolic Composition and Sensory Attributes
Source: Molecules. 2023 Oct 10;28(20):7006. doi: 10.3390/molecules28207006 (PMC10609335; doi:10.3390/molecules28207006)
Supplement: Supplementary file 1 [file molecules-28-07006-s001.zip › molecules-2548923-supplementary.pdf]

Table S1–Pearson Correlation between the sensory descriptors and the acceptance of DG

	BC	OP	IR	DCO	MCO	FO	SW	ST	BT	DCF	MCF	RF	FT	SF	AD	T	P	N	INT	D	ACT
BC	1																				
OP	0.071	1																			
IR	-0.212	0.206	1																		
DCO	0.353*	0.223	0.116	1																	
MCO	-0.072	-0.022	0.356*	-0.192	1																
FO	-0.171	-0.204	0.193	**-0.343***	0.275	1															
SW	0.067	0.071	0.289*	-0.022	0.410**	0.018	1														
ST	-0.095	-0.106	0.060	-0.008	0.240	0.059	-0.029	1													
BT	0.038	-0.117	0.350*	0.409**	0.102	0.023	-0.036	0.116	1												
DCF	0.117	-0.047	0.178	0.472**	-0.048	-0.219	0.012	0.347*	0.489**	1											
MCF	0.073	0.005	0.148	-0.116	**0.699****	0.227	0.447**	0.034	-0.098	**-0.341***	1										
RF	0.007	-0.050	0.332*	0.057	0.158	0.240	0.281	0.361*	0.161	0.299*	0.128	1									
FT	-0.119	-0.219	0.333*	-0.090	0.488**	0.652**	0.021	0.145	0.348*	-0.002	0.369**	0.343*	1								
SF	0.057	-0.018	0.238	0.084	0.297*	0.132	0.009	0.114	0.032	0.197	0.190	0.093	0.189	1							
AD	0.006	-0.025	0.385**	0.194	0.226	0.251	0.330*	-0.032	0.471**	0.106	0.221	0.313*	0.373**	-0.015	1						
T	0.101	0.194	0.317*	0.334*	0.063	-0.051	0.100	0.110	0.188	0.189	0.056	-0.110	-0.155	0.353*	0.092	1					
P	0.332*	0.075	-0.023	0.098	-0.077	0.022	-0.046	-0.048	0.178	0.006	-0.035	-0.049	-0.129	0.079	0.019	0.487**	1				
N	0.050	0.211	0.350*	0.043	0.321*	0.116	0.222	0.139	-0.009	-0.039	0.404**	0.115	0.065	0.457**	-0.021	0.456**	0.317*	1			
INT	0.291*	0.163	0.015	0.134	0.145	0.050	-0.117	0.041	0.122	-0.057	0.193	-0.143	0.011	0.273	-0.066	0.400**	**0.764****	0.403**	1		
D	0.276	0.089	0.207	0.298*	0.022	-0.198	-0.015	0.071	0.236	0.190	-0.053	-0.187	**-0.303***	0.320*	-0.015	**0.751****	**0.606****	0.404**	0.463**	1	
ACT	0.251	-0.020	-0.024	0.137	-0.078	0.006	-0.251	-0.059	-0.042	0.083	-0.087	-0.206	-0.086	**0.440****	-0.196	**0.380****	**0.289***	**0.301***	**0.382****	**0.366****	1

Abbreviations: BC, Brown Color; OP, Opacity; IR, Icing roughness; DCO, Dark chocolate odor; MCO, Milk chocolate odor; FO, Fat/Cocoa butter odor; SW, Sweet taste; ST, Sour taste; BT, Bitter taste; DCF, Dark chocolate flavor; MCF, Milk chocolate flavor; RF, Raisins flavor; FT, Fat/Cocoa butter /Butter taste; SF, Soft texture; AD, Adhesive texture; T, Tasty; P, Pleasant; N, Natural; INT, Interesting; D, Delightful; ACT, Global acceptance; * Significant correlation ($p \leq 0.05$). ** $p < 0.01$ Adapted from Olivati, 2020 [17].
